# Editorial: Waste to wealth: A sustainable circular bioeconomy approach

**DOI:** 10.3389/fbioe.2022.1010811

**Published:** 2022-09-30

**Authors:** Kumar Pranaw, Lukasz Drewniak, Lata Nain, Surender Singh

**Affiliations:** ^1^ Department of Environmental Microbiology and Biotechnology, Faculty of Biology, Institute of Microbiology, University of Warsaw, Warsaw, Poland; ^2^ Division of Microbiology, ICAR-Indian Agricultural Research Institute, New Delhi, India; ^3^ Department of Microbiology, Central University of Haryana, Mahendergarh, India

**Keywords:** circular economy, biorefinery, bioenergy, waste utilization, valorization, sustainable development, biobased chemical

As a consequence of industrialization, global urbanization, and economic development, solid waste generation is increasing rapidly, which requires immediate and effective solutions. Several scientific efforts in response to this challenge resulted in the production of valuable industrial products from waste materials, which is a key factor for the emerging circular bioeconomy concept (Leong et al., 2021). Environmental friendly utilization of lignocellulosic biomass and/or waste materials for the production of value-added products often involves the use of biotechnology. The microbial enzymes convert the lignocellulosics into C6 and C5 sugars via enzymatic hydrolysis. Further, these sugars can be converted into targeted bio-products by specific microorganisms. Utilization of lignocellulosics for these value-added products will not only reduce the dependency on fossil fuels but will also help in environment friendly production of value-added products e.g., chemicals (like organic acids, furans, polyols, oligosaccharides, polyhydroxyalkanoates, etc.), or other bio-products (like phenolic compounds, pigments, fertilizers, etc.) (Grewal et al., 2022; Singh et al., 2022).

This Research Topic on *Waste to wealth: A sustainable circular bioeconomy approach* provided a platform for novel research that aims to suggest new multidisciplinary theoretical and experimental designs for the process of utilizing agro-industrial waste materials for biorefineries by extracting not just bioenergy and/or biofuels but also other valuable platform chemicals, oligomers, and polymers.

The research focus of all the accepted papers was mainly the use of wild-type as well as genetically engineered microbes for the production of industrially important chemicals by utilizing agro-industrial wastes. The first research paper was mainly focused on the production and characterization of cellulases from *Bacillus subtilis* CD001 which was acidothermophilic in nature and might find widespread industrial applications in biomass saccharification (Malik and Javed). Another article by Jocquel et al. was engrossed on transglycosylation reaction using commercial enzymatic cocktail Cellic Ctec2 in the presence of pentanol which led to the synthesis of pentyl β-D-xylosides using xylans enzymatically derived from wheat bran. Another article by Zerva et al. again focused on *β-Glucosidase and β-Galactosidase-Mediated Transglycosylation of Steviol Glycosides Utilizing Industrial Byproducts.* In this study, the role of glycosyl hydrolases (i.e., β-glucosidase, MtBgl3a, and a β-galactosidase, TtbGal1) in the transglycosylation reaction of different steviol glycosides (mainly stevioside and rebaudioside A) was deciphered for the improvement of their taste as an artificial sweetener. To achieve this goal, they employed different low-cost industrial byproducts as sugar donors, such as cellulose hydrolyzate and acid whey for TtbGal1-and MtBgl3a-mediated bioconversion, respectively.

Further three papers focus on genetically engineered bacteria for different industrial applications. Burgardt et al. investigated the production of l-lysine-derived bifunctional monomers using metabolically engineered *Corynebacterium glutamicum* strains using wheat sidestream concentrate (WSC), a byproduct from the industrial starch production industry. They deciphered that heterologous expression of the genes *xyl*AXcBCg (*xyl*A from *Xanthomonas campestris*) and *ara*BADEc from *Escherichia coli*, along with supplementation of xylose and arabinose in WSC hydrolysate (WSCH) would increase the production of l-lysine. For the production of cadaverine and 5-aminovalerate (5AVA) using WSCH, the lysine decarboxylase gene *ldc*CEc from *E. coli* was expressed for the conversion to 5AVA cascaded either with putrescine transaminase and dehydrogenase genes *pat*DAEc or with putrescine oxidase gene *puo*Rq from *Rhodococcus qingshengii* and *pat*DEc. In another article, Tiwari et al., successfully explored the catabolic pathway of levulinic acid (LA) in the genetically engineered *Pseudomonas putida* and engineered this strain for the sustainable production of propionic acid. Primarily, it was achieved by deleting the methylcitrate synthase (*Prp*C) and propionyl-CoA synthase (*Prp*E) genes in *P. putida* EM42 strain. Subsequently, a LA-inducible expression system was employed to express *yci*A (encoding thioesterase) from *H. influenzae* and *ygf*H (encoding propionyl-CoA: succinate CoA transferase) from *E. coli* to improve the propionic acid production by up to 10 folds.

Sathesh-Prabu et al. developed a levulinic acid (LA)-inducible and antibiotic-free plasmid system to produce highly beneficial, large-scale cost-effective microbial production of value-added products like 4-hydroxyvaleric acid from LA derived from renewable substrates. To achieve their goal, an engineered *E. coli* strain was developed by engineering the 5′ untranslated regions (UTR) of *hpd*R mRNA, and expressing the engineered 3-hydroxybutyrate dehydrogenase (3HBDH*) and formate dehydrogenase (CbFDH). Upscaling this process at a 5-L Fermenter level resulted in 82 g/L of 4-HV from LA in the fed-batch fermentation without adding antibiotics and external inducers. In summary, the articles published in this Research Topic mainly focused on the exploitation of microbial strains to generate high titer of unique metabolic byproducts by valorizing agro-industrial waste and side streams advocating a sustainable circular bioeconomy.
